# Tofersen Treatment in *SOD1*-ALS: Real-World Evidence from a Retrospective Multicenter Study in France (FORSLA Study)

**DOI:** 10.1016/j.mayocpiqo.2026.100739

**Published:** 2026-07-27

**Authors:** Daniells Erazo, Adele Hesters, Gaëlle Bruneteau, Elisa De La Cruz, Emilien Bernard, Aude-Marie Grapperon, Gwendal Le Masson, Pascal Cintas, Marie-Hélène Soriani, Nathalie Guy, Julien Cassereau, Jean-Philippe Camdessanché, Steeve Genestet, Philippe Corcia, Sophie Pittion, Véronique Danel, Mathilde Filliatre, Ariane Choumert, Agnes Jacquin-Piques, François Salachas, Florence Esselin, Adrien Bohic, Géraldine Lautrette, Shahram Attarian, Julie Catteau, Claire Guissart, Marie-Claire Vincent, Kevin Mouzat, Marie Temple, Patrick Vourc’h, Philippe Couratier

**Affiliations:** a FILSLAN – French rare disease healthcare network, France; b Inserm U1094, IRD UMR270, University of Limoges, University Hospital Limoges, EpiMaCT - Epidemiology of chronic diseases in tropical zone, Institute of Epidemiology and Global Health – Michel Dumas, OmegaHealth, Limoges, France; c ALS and other motor neuron disease reference center, University Hospital Limoges, France; d ALS and other motor neuron disease reference center, University Hospital Pitié-Salpêtrière, Assistance Publique – Hôpitaux de Paris, Paris, France; e University of Paris Cité and Inserm UMR_S1124, Paris, France; f Alliance on Clinical Trials for ALS-MND (ACT4ALS-MND), Neuroscience Clinical Investigation Center, Paris Brain Institute, Paris, France; g Institute for Neurosciences of Montpellier (INM), University of Montpellier, INSERM, France; h ALS and other motor neuron disease reference center, University Hospital Montpellier, France; i ALS and other motor neuron disease reference center, Hospices Civils de Lyon, France; j Institut NeuroMyoGène, CNRS UMR5310, INSERM U1217, Faculty of Medicine Rockefeller, Claude Bernard Lyon I University, Lyon, France; k ALS and other motor neuron disease reference center, Euro-NMD ERN, Assistance Publique – Hôpitaux de Marseille, Timone University Hospital, Marseille, France; l ALS and other motor neuron disease reference center, University Hospital Bordeaux, France; m INSERM U1215, University of Bordeaux, France; n ALS and other motor neuron disease reference center, University Hospital Toulouse, France; o University Nice Cote d’Azur UR2CA-URRIS, France; p ALS and other motor neuron disease reference center, University Hospital Nice, France; q ALS and other motor neuron disease reference center, University Hospital Clermont-Ferrand, France; r Inserm U 1107, Neuro-dol, University of Clermont-Ferrand, France; s ALS and other motor neuron disease reference center, University Hospital Angers, France; t MITOVASC UMR CNRS 6015-INSERM U1083, Equipe Mitolab, University of Angers, France; u ALS and other motor neuron disease reference center, University Hospital Saint-Etienne, France; v Department of Neurology, University Hospital Saint-Etienne, France; w ALS and other motor neuron disease reference center, University Hospital Brest, France; x Inserm Unit U1253, team IdeALS, University of Tours, France; y ALS and other motor neuron disease reference center, University Hospital Tours, France; z European Reference Network for Rare Neuromuscular Diseases (EURO-NMD); aa ALS and other motor neuron disease reference center, University Hospital Nancy, France; bb ALS and other motor neuron disease reference center, University Hospital Lille, France; cc ALS and other motor neuron disease reference center, University Hospital Caen, France; dd ALS and other motor neuron disease reference center, University Hospital Sud Réunion, France; ee University of Bourgogne Europe, University Hospital Dijon Bourgogne, University department of Neurology, Unit of clinical neurophysiology, Dijon, France; ff ALS and other motor neuron disease reference center, University Hospital Dijon, France; gg INM, University of Montpellier, INSERM Montpellier, Department of Biochemisty and Molecular Biology, University Hospital Nîmes, France; hh Department of Medical Genetics, University Hospital Pitié-Salpêtrière, Assistance Publique – Hôpitaux de Paris, Sorbonne University, Paris, France; ii University of Tours, INSERM, Imaging Brain & Neuropsychiatry iBraiN U1253, Tours, France; jj University Hospital Tours, and Department of Medical Genetics, University, SLA reference laboratory (Laboratoire de Biologie Médicale de Référence (LBMR) sur la SLA), Tours, France

## Abstract

**Objective:**

To evaluate the effectiveness of Tofersen in patients with superoxide dismutase 1 gene (*SOD1-ALS*) patients in France in a real-world setting, using disease progression within patient comparisons and with a historical cohort.

**Patients and Methods:**

Patients with *SOD1*-ALS were included from across 19 French FILSLAN network centers. Baseline was defined as the treatment initiation date. Main endpoints were the ALSFRS-R progression rate and plasmatic neurofilament light chain (NfL) levels at baseline and 12 months after baseline.

**Results:**

In the Tofersen Cohort (N=46), within-group comparisons showed that the mean ALS functional rating scale revised (ALSFRS-R) progression rate slowed from 0.53 ± 0.5 at baseline to 0.22 ± 0.3 point/month at 12 months (*P*=.006). NfL levels significantly decreased from 89.0 ± 9.0 pg/ml at baseline to 29.2 ± 19.5.5 at 12 months (*P*=.004). Exploratory comparisons with a propensity score (PS) matched historical cohort (39 matched pairs) using a mixed-effects model, ALSFRS-R progression rate at baseline, 6 months, and 12 months after baseline, showed no statistically significant differences between groups *P*=.30, whereas longitudinal ALSFRS-R scores differed significantly between groups (time-treatment interaction *P*=.006). The mean survival of the PS matched population was longer in the Tofersen Cohort (42.6 months) than the Historical Cohort (31.8 months) *P*=.004. Time-dependent adjusted cox analysis showed that Tofersen was associated with a reduction in mortality risk (adjusted HR=0.34; 95% CI, 0.12-0.91; *P*=.03).

**Conclusion:**

Tofersen seems to be associated with slower functional decline and reduced NfL levels. While limitations of retrospective design and ALSFRS-R sensitivity must be acknowledged, these findings provide real-world evidence suggesting a clinical benefit of Tofersen.

Amyotrophic lateral sclerosis (ALS) is a fatal neurodegenerative disease characterized by progressive muscle weakness that leads to limb paralysis, dysphagia, dysarthria, and ultimately respiratory failure.^1^ Median survival is 3 years from symptom onset.^1^ ALS has an incidence in France of around 2.5 per 100,000^2^^,^^3^, ∼10% to 15% of cases are familial while the remaining 85% to 90% of cases are sporadic.^4^ ALS associated with a pathogenic variant in the superoxide dismutase 1 gene (*SOD1*-ALS) accounts for ∼1.6% of ALS cases in France, classifying it as an ultra-rare disease.^5^ Disease progression in *SOD1*-ALS is heterogeneous, ranging from rapidly progressive forms to more slowly progressing phenotypes.

There is currently no cure for *SOD1*-ALS. In France, riluzole is the only treatment commercially available but has a modest effect and only delays the progression of the disease by 2 to 3 months.^6^ In recent years, new molecular therapies have been developed to target ALS genetic mutations and neutralize their toxic products.^7^ Among them, the antisense oligonucleotide Tofersen, administered by intrathecal injections, was approved by the European Medicines Agency in May 2024 for the treatment of adults with *SOD1*-ALS. Approval was based on the results of the Phase 3 VALOR clinical trial and its open-label extension (OLE) phase.^8^ Although the primary endpoint was not met (no improvement in the ALS functional rating scale revised (ALSFRS-R) score after 28 weeks of treatment), the trial reported a considerable decrease in neurofilament light (NfL) level, a biomarker of ALS progression in plasma compared to placebo and a greater reduction in concentrations of the SOD1 protein in the cerebrospinal fluid. In addition, a slowing of ALSFRS-R decline was reported during the OLE stage.

In France, Tofersen has been available since February 2022 for patients with *SOD1*-ALS, as part of a Compassionate Use Program (CUP), granted by the French Health Authority (HAS). As the primary endpoint of the VALOR trial was not met, the HAS did not approve tofersen for the treatment of *SOD1*-ALS.

In this context, the FILSLAN rare disease healthcare network conducted a retrospective multicenter study that aimed to evaluate the effectiveness of tofersen in routine clinical practice on both clinical progression and NfL change and to explore the potential benefit compared with standard of care (SOC).

## Patients and Methods

### Study Design

This study involved 19 French ALS expert centers, affiliated with the FILSLAN rare disease healthcare network (Filière de santé SLA et Maladies du Neurone moteur). Molecular biology laboratories from the network first identified all adult patients with ALS, diagnosed according to the revised El Escorial criteria and carrying a pathogenic *SOD1* variant (class 4 or 5, as defined by the American College of Medical Genetics and Genomics [ACMG]). Identified patients were then included in 2 cohorts: the Tofersen Cohort (ie, patients treated with Tofersen intrathecally 100 mg every 28 days [and riluzole]) and the Historical Cohort (ie, patients treated with riluzole 50 mg twice a day per SOC), according to the criteria outlined in the participants’section.

For the tofersen cohort, data were obtained from the tofersen CUP database and the patients’ medical charts, while data for the historical cohort were extracted from the national ALS database of the French Motor Neuron Disease Study Group. When entries were missing or incomplete in the database, the referring neurologists at each center completed the information from the patients’medical charts. All extracted data was confirmed by each referring center to assure data quality.

For each patient, data of interest were collected at symptom onset, diagnosis, at baseline (treatment initiation) at 6 months and 12 months following treatment initiation. Other information included were vital status at last visit, date of the patient's death or loss to follow-up. The observation period ended on April 30, 2025 for all enrolled patients. Baseline was defined as the date of the first administration of tofersen in the tofersen cohort and the date of first administration of riluzole in the historical cohort.

### Participants

As per French regulation, patients with *SOD1*-ALS still alive at study initiation were informed of the study and provided with a patient information and nonopposition note. Patients who did not receive the note, who objected to the reuse of their data for research purposes, or who died before data collection began and who, during their lifetime, objected to the use of their data were excluded from the study.

Patients were then selected for each cohort according to the following main criteria. For inclusion in the tofersen cohort, patients had to have initiated treatment with tofersen and received their first dose from February 1, 2022 to July 31, 2024 as part of the CUP. All patients in the tofersen cohort received riluzole as this is part of the SOC for all ALS patients in France. In addition, patients were required to have completed at least six months of treatment with tofersen before the end of the observation period. For inclusion in the historical cohort, patients were required to have been diagnosed from January 1, 2008 to July 31, 2024 and to have been treated with riluzole and not received any tofersen treatment.

### Data Collection

Collected data included demographics, history of the disease (symptom onset, diagnosis, and initial site of motor impairment), diagnosis delay (calculated as the time in months between the date of first symptoms and date of diagnosis), outcomes at 6-months and 12-months post-treatment (including clinical progression and plasma NfL levels), and adverse events of special interest (ie, myelitis, aseptic meningitis, meningeal pleocytosis, and intracranial hypertension). Plasmatic NfL levels were measured by a centralized accredited laboratory.

For the evaluation of clinical progression, ALSFRS-R scores were therefore collected at diagnosis, baseline, 6 months, and 12 months post-treatment. ALSFRS-R is a 48-point clinical scale assessing functional impairment across bulbar, fine motor, gross motor, and respiratory domains. ALSFRS-R progression rate is defined as the number of ALSFRS-R points lost per month.

### Outcomes

The primary outcome was the efficacy of tofersen in patients with *SOD1*-ALS, assessed by the change in ALSFRS-R progression rate at 12 months of treatment compared with baseline. Secondary outcomes included: (1) change in ALSFRS-R progression rate at 6 months; (2) change in total ALSFRS-R score at 6 and 12 months; (3) change in plasma NfL levels at 6 and 12 months; (4) adverse events of special interest associated with tofersen; (5) comparison of tofersen with SOC based on ALSFRS-R progression rate; and (6) comparison of tofersen with SOC based on survival at 48 months after diagnosis.

ALSFRS-R progression rates were calculated for the study endpoints with predefined formulas. At baseline, the rate was calculated as (48-ALSFRS-R at treatment initiation)/(Date of treatment initiation—Date of symptom onset); and at 6/12 months of treatment as (ALSFRS-R at 6/12 months—ALSFRS-R at treatment initiation)/(Date of the 6/12 months visit—Date of treatment initiation).

### Statistical Analyses

The sample size was determined by the number of patients meeting eligibility during the study period; no formal calculation was performed.

For subgroup analysis, included patients were classified according to their ALSFRS-R progression rate at diagnosis as follows: slow progressor (<0.30 point/month), intermediate progressor (0.30-0.79 point/month), or fast progressor (≥0.80 point/month) (5). ALSFRS-R progression rate at diagnosis was calculated as (48—ALSFRS-R score at diagnosis)/(time from symptom onset to diagnosis, in months).

Descriptive analysis was performed with continuous variables summarized with mean, SD, median, first and third quartile (Q1, Q3), range, minimum and maximum and categorical variables summarized with frequencies and percentages. Comparisons of continuous variables were performed using the Wilcoxon signed-rank test for paired data and the Mann-Whitney U test for independent groups. Categorical variables were compared using the χ^2^ test or the Fisher's exact test when appropriate.

As the study did not involve random treatment allocation, a PS was estimated to account for potential baseline differences between groups. The PS was calculated using a binary logistic regression model in which treatment status (tofersen vs no tofersen) was the dependent variable. Covariates were selected a priori based on clinical relevance and included sex, genetic variant (homozygous vs heterozygous), age at symptom onset, age at diagnosis, diagnostic delay, ALSFRS-R score at the first visit to an ALS center, time from symptom onset to riluzole initiation, disease progression category at diagnosis (slow/intermediate/fast), and site of onset (bulbar vs spinal). Patients in the tofersen cohort were matched 1:1 with patients from the historical cohort using nearest-neighbor matching without replacement, based on the estimated PS. A caliper of 0.04 on the raw PS scale was applied to restrict acceptable matches. Covariate balance after matching was assessed using SMD, with values <0.10 indicating adequate balance (Supplemental Table 1, available online at http://www.mcpiqojournal.org). All covariates met this criterion, and no additional adjustment was required.

For the Tofersen Cohort only, comparisons between baseline and post-treatment time points (6 and 12 months) were performed for the ALSFRS-R progression rate, ALSFRS-R total score, and NfL levels using the Wilcoxon signed-rank test. To account for multiple comparisons across the six pre-and post-treatment analyses, *P*-values were adjusted using the Holm–Bonferroni method.

Longitudinal changes in ALSFRS-R progression rate and ALSFRS-R score between the tofersen and historical cohorts were analyzed using a mixed-effects model including time, treatment group, and their interaction. The *P* value for the time-treatment interaction was used to assess whether the change in ALSFRS-R progression over time differed between the two cohorts. For analyses performed in the overall populations, models were adjusted for clinically relevant covariates selected *a priori* based on their known prognostic value in ALS, including: age at diagnosis, diagnosis delay, time between diagnosis and baseline, sex, site of onset, ALS progression rate at baseline and ALSFRS-R total score at baseline. For analyses performed in the fast progressor subgroup and in the PS matched population, adjustment was limited to ALSFRS-R progression rate at baseline, ALSFRS-R total score at baseline, and time between diagnosis and baseline because of the reduced sample size and limited number of observations in these subgroups. Estimated marginal means (EMM) with corresponding SE, as well as β estimates, 95% CIs, and p-values, were reported. Analyses were performed in the overall population, in the fast progressor subgroup, and in the PS matched population.

Survival was analyzed using Kaplan–Meier curves with survival defined as the time from diagnosis to reported death, with censoring at 48 months after diagnosis. Patients who were alive at the end of the post-baseline period or lost to follow-up were censored at the date of last contact. To address potential immortal time bias related to the delay between diagnosis and treatment initiation, exposure to tofersen and riluzole was modeled as a time-dependent variable in cox proportional hazards model analyses.^9^ In the overall population, models were adjusted for known prognostic factors in ALS, including treatment group, age at diagnosis, ALSFRS-R progression rate at diagnosis and ASLFRS-R score at diagnosis, sex, diagnosis delay, and ALS site of onset. For analyses performed in the PS matched population, the number of adjustment covariates was reduced because of the limited sample size and number of death events. Analyses were performed in the overall *SOD1-ALS* population and in the PS matched population.

Statistical analyses were performed using SPSS v. 28, JASP v. 0.95.1.0 and Rstudio (R version 4.6.0). All statistical tests were two-tailed, with the significance level set at 0.05.

### Institutional Review Board

This retrospective, noninterventional study used data from previously declared databases. A summary of the study was registered on the Health Data Hub platform N° 24859945.

All patients were individually informed through an information notice describing the objectives, nature, and modalities of data processing. In accordance with current regulations, patients had the right to object to the use of their data for research purposes.

Data sharing and use agreements were established between Limoges University Hospital and each participating center.

## Results

### Disposition and Participant Characteristics

**Figure 1:**
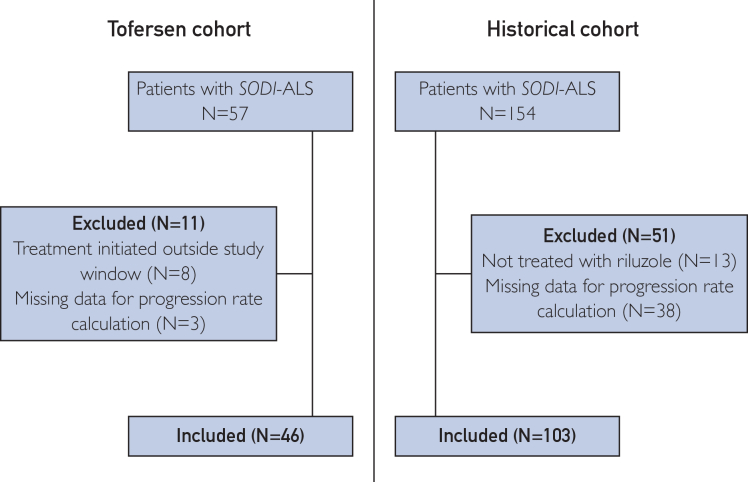
Participant selection. *SOD1*-ALS, amyotrophic lateral sclerosis associated with a SOD1 pathogenic variant.

Two hundred and eleven *SOD1*-ALS patients were identified by the molecular biology laboratories of the FILSLAN network. After applying the eligibility criteria, 103 patients were included in the Historical Cohort and 46 patients in the tofersen cohort (Figure 1).

The mean age of patients in the tofersen cohort was 54.5 years at symptom onset and 55.5 years at diagnosis (Table 1). Bulbar-onset forms were more frequent in the historical cohort 10 (9.7%) compared with the tofersen Cohort 1 (2.2%) (*P*=.05). ALSFRS-R progression rates at diagnosis showed a relatively even distribution of fast, intermediate, and slow progressors within the tofersen cohort. In contrast, in the historical cohort there was a higher proportion of patients with intermediate 42 (42%) or fast 38 (38%) progression forms of the disease, although this difference was not statistically significant. Characteristics of the matched population are presented in Table 2.

**Table 1:** Patient Characteristics

Characteristics	Overall (N=149)	Tofersen cohort (n=46)	Historical cohort (n=103)	*P* value^a^
Demographics				
Female, n (%)	75.0 (50.3)	26.0 (56.5)	49.0 (47.6)	.40
Age at symptom onset (y), Median (IQR)	56.0 (45.5-61.5)	54.5 (43.2-59.7)	57 (48.0-64.0)	.16
Missing	2	0	2	
Age at diagnosis (y), Median (IQR)	57.0 (47.0-65.0)	55.5 (45.0-62.5)	58,0 (49.0-65.0)	.34
Missing	0	0	0	
Disease characteristics at diagnosis				
Age at baseline (y), Median (IQR)	57.0 (49.0-65.0)	56.5 (47.2-63.2)	58.0 (49.0-66.0)	.75
Diagnostic delay (m), median (IQR)		15.7 (6.2-31.3)	13.9 (7.3-23.3)	.72
ALS onset, n (%)				.05
Bulbar	11 (7.4)	1 (2.2)	10 (9.7)	
Spinal				
Cervical	21 (14.1)	3 (6.5)	18 (17.5)	
Thoracic	4 (2.7)	1 (2.2)	3 (2.9)	
Lumbar	109 (73.2)	41 (89.1)	68 (66.0)	
Respiratory	4 (2.7)	0	4 (3.9)	
ALSFRS-R, Median (IQR)	40.0 (35.0-43.0)	41 (36.0-44.0)	40 (34.2-43.0)	.67
Missing	0	1	1	
ALSFRS-R slope since onset, median (IQR)	0.53 (0.3-1.1)	0.44 (0.2-1.1)	0.55 (0.3-1.1)	.23
ALSFRS-R progression, n (%)				
Slow	36.0 (24.8)	16 (35.6)	20 (20.0)	.12
Intermediate	56.0 (38.6)	14 (31.1)	42 (42.0)	
Fast	53.0 (36.6)	15 (33.3)	38 (38.0)	
Missing		1	2	
Disease characteristics at baseline				
Time between diagnosis and baseline (m), median (IQR)	0.7 (0.0-5.3)	7.7 (5.2-35.9)	0.0 (0.0-0.8)	-
ALSFRS-R score, median (IQR)	38.0 (33.0-43.0)	35.0 (31.0-41.0)	40.0 (34.2-43.0)	.01
ALSFRS-R slope since onset, median (IQR)	0.49 (0.2-0.9)	0.3 (0.2-0.7)	0.5 (0.3-1.1)	.001

ALS, amyotrophic lateral sclerosis; ALSFRS-R, ALS functional rating scale – revised; IQR, interquartile range; m: months; SD, standard deviation; y: years.

a Statistical comparisons were performed to evaluate the differences between the groups (Tofersen vs Historic). A Mann-Whitney U test was used for continuous variables and a Fisher’s exact test was used for categorical variables.

**Table 2:** Sociodemographic, Genetic, and Clinical Characteristics in the PS Matched Population

Characteristics	Tofersen (n=40)	Historic (n=40)	*P* value	SMD
Demographics				
Sex, n (%)				
Male	18.0 (45.0)	20.0 (50.0)	.82	0.10
Female	22.0 (55.0)	20.0 (50.0)		
Sex ratio	0.9	1.0		
Age at symptom onset (Y). Median (IQR)	56.0 (44.0-60.7)	55.0 (43.0-64.2)	.95	0.01
Age at diagnosis, (Y), Median (IQR)	57.0 (45.0-64.0)	56.0 (44.0-66.0)	.98	0.02
Diagnostic delay (m), median (IQR)	15.7 (6.8-32.9)	17.4 (9.5-27.2)	.51	0.06
Genetic characteristics				
Homozygote	3 (7.5)	4 (10.0)	>.99	−0.09
Heterozygote	37 (92.5)	36 (90.0)		
Disease characteristics at diagnosis				
ALS onset, (%)				
Bulbar	1 (2.5)	1 (2.5)	.75	0.00
Spinal	39 (97.5)	39 (97.5)		
ALSFRS-R score, median (IQR)	39.0 (34.2-43.5)	41.0 (36.0-43.0)	.80	−0.09
ALSFRS-R slope since onset to diagnosis	0.46 (0.2-0.9)	0.40 (0.1-0.4)	.56	0.16
ALSFRS-R progression (%)				
Slow	13.0 (32.5)	13.0 (32.5)	>.99	0.00
Intermediate	14.0 (35.0)	14.0 (35.0)		
Fast	13.0 (32.5)	13.0 (32.5)		
Delay between symptom onset and initiation of riluzole, (m) median IQR	0.4 (0.0-1.0)	0.03 (0.0-1.0)	.77	0.04
Disease characteristics at baseline				
Age at baseline (Y), Median (IQR)	57.0 (47.7-64.5)	56.0 (46.7-67.0)	.563	0.16
ALSFRS-R score, median (IQR)	35 (30.0-41.0)	39.0 (34.0-43.0)	.02	−0.58
ALSFRS-R slope since onset to baseline	0.3 (0.2-0.7)	0.4 (0.2-0.8)	.59	−0.24
Time between diagnosis and baseline, median (IQR)	6.7 (5.2-31.8)	0.1 (0.0-1.2)	<.001	0.94
Interventions				
Noninvasive ventilation				
Yes, n (%)	13.0 (43.3)	19.0 (54.3)	.38	−0.22
Missing	10	5		

Chi2 test; Fisher exact test; Mann-Whitney U test.

Noninvasive ventilation was more frequent in the historical cohort n=55 (60.4%) than in the tofersen cohort n=14 (38.9%) (*P*=.04). No invasive ventilation was performed in the tofersen cohort.

Among the *SOD1*-ALS population, 79 different *SOD1* pathogenic variants were observed and are presented in Supplemental Table 2, available online at http://www.mcpiqojournal.org. The most frequent variant was Asp91Ala (tofersen cohort: n=3; historic cohort: n=8) and patients characteristics carrying the Asp91Ala variant are presented in Supplemental Table 3, available online at http://www.mcpiqojournal.org.

Patients in the tofersen cohort received in average 19 doses (±11) of treatment during the observation period. Among them, 13 discontinued their treatment during the follow-up period: eight by patient decision, three due to the occurrence of serious adverse events, and two due to nonserious adverse events.

### Clinical Outcomes

**Figure 2:**
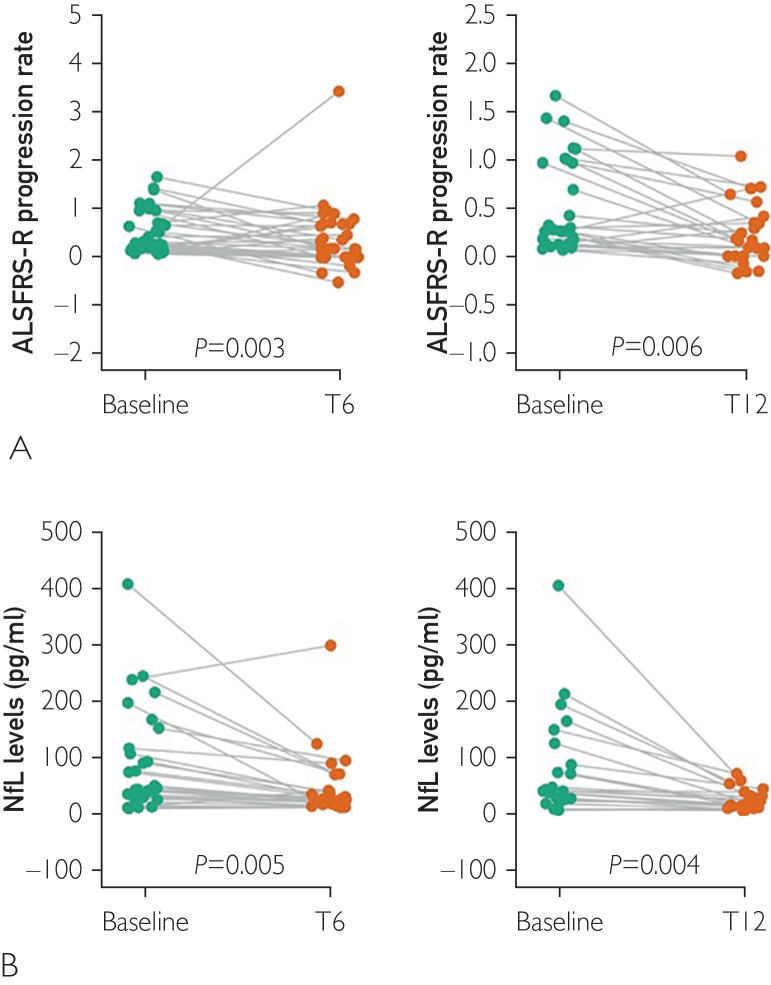
Change in ALSFRS-R progression rate and neurofilament light chain levels at 6- and 12-month post-treatment. Tofersen Cohort. ALS, amyotrophic lateral sclerosis; ALSFRS-R, ALS functional rating scale – revised; NfL, neurofilament light chain; T6, 6 months post-treatment; T12, 12 months post-treatment. (A) ALSFRS-R progression at baseline (n=45), 6-month post-treatment (n=39), and 12-month post-treatment (n=28). (B) Plasma NfL levels at baseline (n=38) and 6 months (n=35). B. Plasma NfL levels at baseline (n=38) and 12 months (n=25).

**Figure 3:**
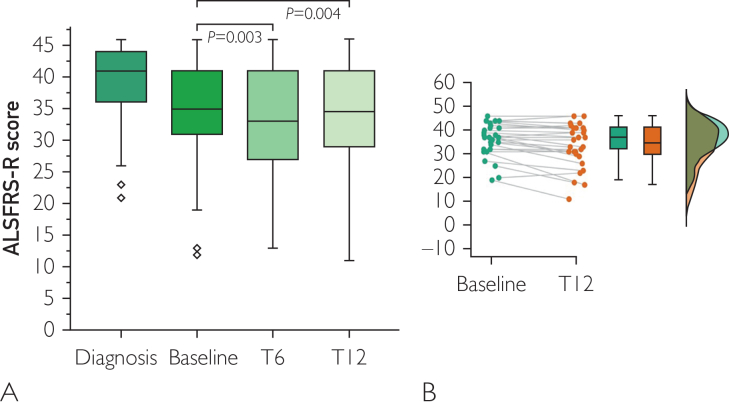
Change in ALSFRS-R score in the Tofersen cohort. ALS, amyotrophic lateral sclerosis; ALSFRS-R, ALS functional rating scale – revised; T6, 6 months post-treatment; T12, 12 months post-treatment. (A) ALSFRS-R score at diagnosis and baseline (n=45), 6-month post-treatment (n=39), and 12-month post-treatment (n=28). (B) Individual change in ALSFRS-R score from baseline to 12 months.

When considering the tofersen cohort only, the mean ALSFRS-R progression rate had significantly reduced from 0.53±0.5 point/month at baseline to 0.22±0.3 point/month after 12 months of treatment (Holm-adjusted *P*=.006). A significant reduction could also be observed at 6 months (0.32 ± 0.6 point/month; Holm-adjusted *P*=.003) (Figure 2A). Consistent with these findings, mean NfL levels had markedly declined from 89.0±9.0 pg/mL at baseline to 41.8±52.5 pg/mL at 6 months (Holm-adjusted *P*=.005) and 29.2±19.5 pg/mL at 12 months (Holm-adjusted *P*=.004) (Figure 2B) while mean ALSFRS-R total score decreased modestly from 34.2±8.2 at baseline to 33.5±9.1 after 12 months of treatment (Holm-adjusted *P*=.004) (Figure 3).

**Figure 4:**
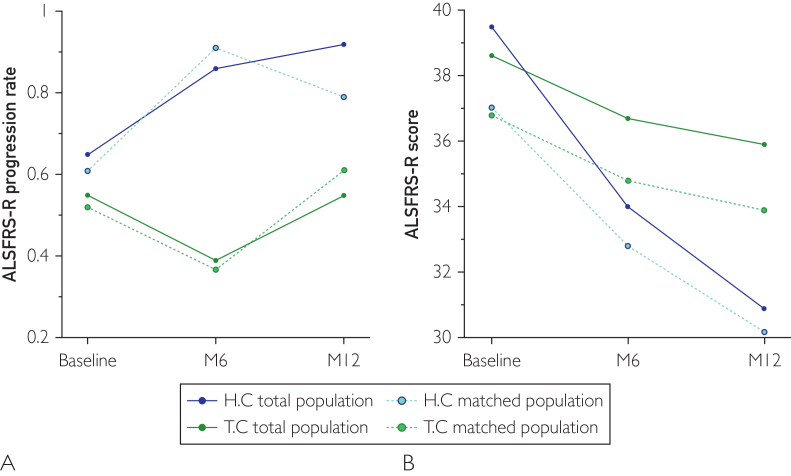
Comparison of ALSFRS-R progression rate and score between the Tofersen and the Historical Cohort in the overall and PS matched population. ALS, amyotrophic lateral sclerosis; ALSFRS-R, ALS functional rating scale - revised; M6, 6 months post-treatment; M12, 12 months post-treatment. (A) Estimated marginal means of ALSFRS-R progression rate, at baseline, 6 months and 12 months. Overall population: Historic cohort (n=100/69/65) Tofersen cohort (n=45/39/28). PS matched population: Historic cohort (n=39/27/26) Tofersen cohort (n=39/34/23). (B) Estimated marginal means of the ALSFRS-R score at baseline, 6 months and 12 months. Overall population: Historic cohort (n=100/69/65) Tofersen cohort (n=45/39/28). PS matched population: Historic cohort (n=39/27/26) Tofersen cohort (n=39/34/23).

When comparing ALSFRS-R progression rates over time between the tofersen and historical cohorts in the overall population (n=149) using adjusted mixed-effects models, estimated marginal means (EMM±SE) in the tofersen cohort were 0.55±0.2 point/month at baseline, 0.39±0.2 point/month at 6 months, and 0.55±0.2 point/month at 12 months. In the historical cohort, corresponding EMM were 0.65±0.2 point/month at baseline, 0.86±0.2 point/month at 6 months, and 0.92±0.2 point/month at 12 months. However, the time-treatment interaction did not reach statistical significance (*P*=.15; Holm-adjusted *P*=.30) (Figure 4A). In contrast, ALSFRS-R progression rate at baseline was significantly associated with longitudinal progression rates in the mixed-effects model (β=95% CI, 0.83-1.11; *P*<.001) (Table 3). Similarly, in the fast progressor subgroup, adjusted mixed-effects analyses did not report a statistically significant time-treatment interaction for ALSFRS-R progression rates (*P*=.06; Holm-adjusted *P*=.18). Baseline ALSFRS-R progression rate also remained significantly associated with longitudinal progression during follow-up in this subgroup (β=0.88, 95% CI, 0.68-1.07; *P*<.001) (Table 3).

**Table 3:** Adjusted Linear Mixed-Effects Model Evaluating Longitudinal Evolution of ALSFRS-R Progression Rate

	Total population		Fast progressors		PS matched population	
	Estimation β (IC95%)	*P*	Estimation β (IC95%)	*P*	Estimation β (IC95%)	*P*
Time-treatment interaction	—	.15	—	.06	—	.19
Time	—	.46	—	.99	—	.60
Treatment	—	.007	—	.002	—	.04
Sex	0.04 (−0.14 to 0.22)	.67				
Age at diagnosis	−0.003 (−0.01 to 0.004)	.40				
ALS site onset	—	.64				
Diagnosis delay	−0.002 (−0.007 to 0.003)	.42				
ALSFRS-R score at baseline	0.0002 (−0.016 to 0.017)	.98			−0.007 (−0.028 to 0.014)	.51
ALSFRS-R progression rate at baseline	0.96 (0.83-1.10)	<.001	0.88 (0.68-1.07)	<.001	0.85 (0.59-1.12)	<.001
Time between diagnosis and baseline	0.002 (−0.002 to 0.006)	.32			0.002 (−0.004 to 0.008)	.60

Because of the missing ALSFRS-R data, only 39 matched pairs were available for evaluation on the PS matched population. The EMM progression rates in the tofersen cohort across all timepoints compared with the historical cohort were at baseline (0.52±0.13 vs 0.61±0.13 point/month) 6 months (0.37±0.0.14 vs 0.92±0.15 point/month) and 12 months (0.61±0.15 vs 0.80±0.17 point/month (Figure 4A). However, the time-treatment interaction (*P*=.19; Holm-adjusted *P*=.30) did not reach statistical significance. ALSFRS-R progression rate at baseline was significantly associated with longitudinal progression rates (β=0.85, 95% CI, 0.59-1.12; *P*<.001) (Table 3).

The same adjusted mixed-effects model was applied to longitudinal ALSFRS-R scores. In the overall population, EMM of ALSFRS-R scores in the historical cohort were 39.5±1.21 points at baseline and 30.9±1.25 points at 12 months. In the tofersen cohort, EMM were 38.6±1.4 points at baseline and 35.9±1.5 points at 12 months (Figure 4B). The time-treatment interaction was statistically significant in the overall population (*P*<.001; Holm-adjusted *P*≤.001). ALSFRS-R score and progression rate was significantly associated (β=0.95, 95% CI, 0.84-1.05; *P*<.001 and β=−1.67, 95% CI, −2.53 to −0.84; *P*<.001, respectively) (Table 4). A similar trend was also observed among the fast progressors population as observed in Supplemental Figure 1 available online at http://www.mcpiqojournal.org and Table 4. In the PS matched population, EMM of the ALSFRS-R score decreased from 36.8±0.65 at baseline to 34.8±0.7 at 6 months and 33.9±0.83 at 12 months in the tofersen cohort compared with 37.2±0.65 at baseline to 32.8±0.77 at 6 months and 30.2±0.8 at 12 months in the historical cohort (Figure 4B); with a time-treatment interaction statistically significant (*P*=.006; Holm-adjusted *P*=.006). Similar to the overall population ALSFRS-R score and progression rate at baseline were significantly associated with longitudinal ALSFRS-R scores in the mixed-effects models (Table 4).

**Table 4:** Adjusted Linear Mixed-Effects Model Evaluating Longitudinal Evolution of ALSFRS-R Score in the Overall, Fast Progressors and PS Matched Population

	Total population		Fast progressors		PS matched population	
	Estimation β (IC95%)	*P*	Estimation β (IC95%)	*P*	Estimation β (IC95%)	*P*
Time∗treatment interaction	—	<.001	—	<.001	—	.006
Time	—	<.001	—	<.001	—	<.001
Treatment	—	.003	—	<.001	—	.01
Sex	−0.40 (−1.61 to 0.81)	.51				
Age at diagnosis	0.02 (−0.02 to 0.07)	.34				
ALS site onset	—	.54				
Diagnosis delay	0.02 (−0.01 to 0.06)	.11				
ALSFRS-R score at baseline	0.95 (0.84 to 1.05)	<.001	0.98 (0.81-1.15)	<.001	0.96 (0.85-1.07)	<.001
ALSFRS-R progression rate at baseline	−1.67 (−2.53 to −0.81)	<.001			−1.72 (−3.12 to −0.33)	.01
Time between diagnosis and baseline	−0.002 (−0.03 to 0.03)	.89			−0.004 (−0.04 to 0.03)	.82

Among the overall population (n=146) seven deaths were observed in the tofersen cohort and 62 in the historical cohort. Survival analysis 48 months after diagnosis of the 39 matched patient pairs showed a mean survival time of 42.6 ± 2.0 months in the tofersen cohort compared with 31.8 ± 2.8 months in the historical cohort (*P*=.004; Holm-adjusted *P*=.004). At 20 months, the probability of survival was 86% in the tofersen cohort versus 68% in the historical cohort. The number of deaths among the PS matched population was six for the tofersen cohort and 19 for the historical cohort. All results of survival analysis are presented in Supplemental Figures 2 and 3 available online at http://www.mcpiqojournal.org. In the time-dependent cox proportional hazards model adjusted for diagnosis characteristics (age, ALSFRS-R progression rate, ALSFRS-R score and ALS onset) for the PS matched population, exposure to tofersen was associated with a significantly reduced risk of death (adjusted HR=0.34; 95% CI, 0.12-0.91; *P*=.03; Holm-adjusted *P*=.03). Age at diagnosis (adjusted HR=1.03; 95% CI, 1.00-1.07; *P*=.02) and ALSFRS-R progression rate at diagnosis (adjusted HR=1.58; 95% CI, 1.01-2.45; *P*=.04) were negatively associated with survival. Consistent results were observed in sensitivity analyses conducted in the overall population, showing a reduction in mortality risk associated with tofersen exposure (Supplemental Table 4, available online at http://www.mcpiqojournal.org)

### Safety

Regarding safety data collected for the tofersen cohort only, 18.2% of patients (n=8) experienced an adverse reaction of interest. Reported reactions were considered serious for six patients (6.8%): aseptic meningitis in 4 patients, myelitis in one patient, and intracranial hypertension in one patient. All 6 events resolved without sequelae. In addition, 15 patients presented with meningeal pleocytosis in the absence of clinical symptoms.

## Discussion

In this study, we evaluated the effectiveness of tofersen in patients with *SOD1*-ALS in a real-world setting, without applying the restrictive inclusion and selection criteria of the VALOR trial.^8^ Notably, enrollment in the tofersen CUP was not limited to patients with specific *SOD1* pathogenic variant or predefined disease progression rates. The primary outcome included within comparisons of the tofersen cohort, secondary outcomes were compared with those of patients receiving SOC (ie, riluzole), which represented the natural history of the disease.

Participant characteristics in the 2 cohorts were relatively homogeneous and in line with previous findings.^8^^,^^10^ However, the historical cohort included a higher proportion of fast progressors. This difference likely reflects variations in treatment initiation: while riluzole is received immediately after diagnosis, tofersen was introduced later through a compassionate access program and was initiated progressively as patients became eligible. Consequently, the median time from diagnosis to tofersen initiation was longer (8 months). This delay may have led to an over-representation of more slowly progressive forms in the tofersen cohort. Several characteristics were comparable between cohorts at diagnosis. However, important differences were present at baseline, particularly regarding ALSFRS-R score, ALSFRS-R progression rate, and the interval between diagnosis and treatment initiation. These differences likely reflect the delayed availability of tofersen through the compassionate access program and underline the need for adjusted longitudinal analyses.

PS matching was used to mitigate baseline imbalances between cohorts. In the PS matched population, most measured baseline characteristics were balanced. However, the time between diagnosis and treatment initiation remained longer in the tofersen cohort, with a median of 6.7 months, which could potentially introduce immortal time bias in survival analyses and lead to an overestimation of treatment benefit. In addition, the requirement for a minimum duration of tofersen treatment may have further contributed to selection bias by excluding early fast progressors who may not have survived long enough to continue treatment. To address this issue, treatment exposure was modeled as a time-dependent variable in cox proportional hazards model analyses, allowing the pre-treatment and post-treatment periods to be distinguished. Results from these time-dependent models remained consistent with the main findings supporting the robustness of the observed survival benefit associated with tofersen. Nevertheless, residual bias cannot be fully excluded and should be considered when interpreting these results. It is also possible that the different time frames beginning in 2022 for the tofersen cohort and 2008 for the historical cohort play a role, but it seems unlikely that a change in the SOC between these 2 periods could account for a 67% reduction in the neurofilament level at 12 months in the tofersen cohort. In addition, a study performed using population-based data from the ALS Limousin registry which compared 2 different periods 2000-2010 versus 2011-2020, median survival was slightly longer in period 2011-2020 (17.0 months, 95% CI, 14.7-19.3 months) compared with period 2000-2010 (14.0 months, 95% CI, 11.8-16.1 months), but the difference did not reach statistical significance (*P*=.09).^11^

Our findings seem to show that tofersen considerably slows disease progression. This was reflected by stabilization of the ALSFRS-R score at 6 and 12 months, as well as a reduction in the ALSFRS-R progression rate after 12 months (0.22±0.3 vs 0.53±0.5; *P*<.001). A marked decline in NfL levels at 12 months further supports these results (29.2±19.5 pg/mL vs 89.0±9.0 pg/mL (*P*<.001). Exploratory survival analyses and cox time-dependent model also seemed to indicate improved outcomes in the tofersen cohort compared with the historical COHORT. In adjusted linear mixed-effects models of ALSFRS-R progression rate, the time-treatment interaction was no statistically significant, suggesting that baseline disease trajectory explained an important proportion of longitudinal variability between cohorts. In contrast, the time-treatment interaction for ALSFRS-R total score remained statistically significant in both the overall and PS matched populations, supporting a persistent association between tofersen exposure and slower functional decline over time despite adjustment for baseline disease severity and progression.

Adjusted models showed that ALSFRS-R progression rate at diagnosis and baseline was strongly associated with subsequent longitudinal progression trajectories and survival, highlighting the potential importance of progression rate as a prognostic marker and its possible relevance for personalized treatment strategies, particularly in patients with faster disease progression at diagnosis.

These findings are consistent with results from the extended OLE study over 148 weeks, in which earlier initiation of tofersen, compared with delayed initiation, was associated with numerically smaller declines in clinical and functional outcomes, including ALSFRS-R score (−9.9 vs −13.5 points), respiratory function assessed by slow vital capacity (−13.8% vs −18.1%), muscle strength measured by handheld dynamometry megascore (−0.38 vs −0.43 points), and quality of life measures. Furthermore, tofersen treatment was associated with prolonged survival compared with the natural history of *SOD1-ALS*.^12^

Our results are consistent with those reported by Wiesenfarth et al,^10^ who evaluated 24 patients treated with tofersen in the German early access program over a median observation period of 6 months. In their study, they reported a median ALSFRS-R progression rate of 0.11 point/month during follow-up compared with a progression rate of 0.41 point/month prior therapy. This result was associated with a reduction in median NfL levels from 78.0 pg/mL at baseline to 36.0 pg/mL during the observation period.

Although our study was based on secondary data (with adverse events previously reported) adverse events of special interest were collected. Of note, fifteen cases of asymptomatic meningeal pleocytosis were observed. Similar reactions have been previously described with the use of tofersen^13^^,^^14^ and with other antisense oligonucleotides, such as nusinersen.^15^ Overall, tofersen is well tolerated but careful monitoring of cerebrospinal fluid and clinical status remains essential.

Treatment adherence was generally good; however, eight severely disabled patients discontinued the treatment as they found the intrathecal procedure too burdensome or the perceived benefit insufficient. To address these challenges, the FILSLAN network established multidisciplinary review meetings to validate treatment indications and systematically assess the benefit–risk balance for each patient.

These real-world findings suggest a potential beneficial effect of tofersen in patients with *SOD1-ALS*. However, these results should be interpreted with caution given the limitations inherent to the study design, particularly the differences in the time interval between diagnosis and baseline assessment between cohorts.

This study has several limitations. First as *SOD1*-ALS is an ultra-rare disease, the number of cases was limited. At the time of analysis, only 28 of the 45 patients in the Tofersen cohort had completed 12 months of follow-up. The limited sample size constrained the complexity of the PS model and subsequent matched analyses. In particular, although the time interval between diagnosis and baseline differed substantially between cohorts and represented an important source of heterogeneity, incorporation of this variable directly into the PS model markedly reduced the number of matched pairs and resulted in less balanced matched populations overall. We therefore prioritized clinically relevant prognostic variables measured at diagnosis in order to maximize comparability between cohorts while preserving an adequate matched sample size

The choice of ALSFRS-R progression rate as the primary endpoint was chosen to evaluate the clinical impact of tofersen. However, this patient reported scale is subject to bias and poses challenges for comparison because of the heterogeneous and non-linear nature of ALS progression. Change in NfL level is being recognized as a more reliable outcome measure to evaluate effectiveness as it is a biomarker strongly correlated with ALS progression and survival.^16^^,^^17^ Unfortunately, NfL measurements were not consistently available in the historical cohort, limiting their use for comparative analyses.

In addition, due to the retrospective nature of the study, missing data was high in the historical cohort and led to the exclusion of 51 patients, reducing the ability to match each tofersen-treated patient with a control and limiting the evaluation of potential confounders such as NfL and *SOD1* variant types.

Despite these limitations, our findings provide important real-world evidence suggesting a clinical benefit of tofersen. While initial randomized trials did not report statistically significant efficacy on primary endpoints, accumulating real-world data and biomarker evidence seem to suggest that tofersen positively impacts disease progression in patients with *SOD1*-ALS. Further studies with larger sample size and follow-up data are needed to confirm tofersen efficacy. Overall, tofersen appears to be generally safe, but clinicians should remain vigilant for potential adverse effects and continuously reassess the risk–benefit balance of therapy.

## Potential Competing Interests

Dr Couratier reported links of interest with Biogen. Dr Jacquin-Piques reported links of interest with Novartis, Argenx, UCB, Roche, Lupin, Sanofi, Alexion, Alnylam, AstraZeneca, Teva, Johnson & Johnson, Amgen, Pfizer, and Biogen. Dr De La Cruz reported links of interest with Biogen. Dr Masson reported links of interest with Argenx, CSL Behring and Zambon. The remaining authors report no competing interests.

## Ethics Statement

This retrospective, noninterventional study used data from previously declared databases. A summary of the study was registered on the Health Data Hub platform N° 24859945. No ethical committee approval was required. All patients were individually informed through an information notice describing the objectives, nature, and modalities of data processing. In accordance with current regulations, patients had the right to object to the use of their data for research purposes.
