# Supplementary material for: Tofersen Treatment in SOD1-ALS: Real-World Evidence from a Retrospective Multicenter Study in France (FORSLA Study)
Source: Mayo Clin Proc Innov Qual Outcomes. 2026 Jul 27;10(5):100739. doi: 10.1016/j.mayocpiqo.2026.100739 (PMC13429897; doi:10.1016/j.mayocpiqo.2026.100739)
Supplement: Supplemental Online Material [file mmc1.docx]

**Contents**

Table S1: Standardized Mean Differences of propensity score variables 2

Table S2: *SOD1* pathogenic variants in the overall population 3

Table S3: Sociodemographic, and clinical characteristics of p.Asp91Ala variant ALS-SOD1 patients 5

Figure S1: Comparison of ALSFRS-R progression rate and score between the Tofersen and the Historical Cohort in the fast progressors population 6

Figure S2: Kaplan Meier analysis for the overall population 7

Figure S3: Kaplan Meier Analysis for the matched population 8

Table S4: Adjusted time-dependent Cox model. 9

# Table S1: Standardized Mean Differences of propensity score variables

| **Variable** | **SMD** |
| --- | --- |
| Sex | 0.074 |
| Age at symptom onset | 0.014 |
| Age at diagnosis | 0.023 |
| Diagnostic delay | 0.055 |
| Genetic variant (Homozygous/Heterozygous) | 0.088 |
| ALS onset site | 0.000 |
| ALSFRS-R score | 0.059 |
| ALSFRS-R progression | 0.000 |
| Delay between symptom onset and initiation of riluzole | 0.036 |
| ALS, amyotrophic lateral sclerosis; ALSFRS-R: ALS functional rating scale – revised; SMD, standardized mean difference | |

# Table S2: *SOD1* pathogenic variants in the overall population

|  |  | **Tofersen Cohort**  **%** | **Historic Cohort**  **%** |
| --- | --- | --- | --- |
| **Nucleotic variant** | **Proteic variant [a]** |  |  |
| c.272A>C | p.Asp91Ala | 6.7 | 8.7 |
| c.358-10T>G | p.Val119_Val120insPheLeuGln | 4.4 | 5.8 |
| c.131A>G | p.His44Arg | 2.2 | 4.9 |
| c.65A>G | p.Glu22Gly | 4.4 | 4.9 |
| c.435G>C | p.Leu145Phe | 6.7 | 1.0 |
| c.418A>G | p.Asn140Asp | - | 3.9 |
| c.280G>T | p.Gly94Cys | 8.9 | - |
| c.358G>C | p.Val120Leu | 6.7 | - |
| c.140A>G | p.His47Arg | 2.2 | 1.9 |
| c.260A>G | p.Ans87Ser | 2.2 | 1.9 |
| c.146A>C | p.His49Pro | - | 2.9 |
| c.14C>T | p.Ala5Val | 2.2 | 1.9 |
| c.199C>T | p.Pro67Ser^a^ | - | 2.9 |
| c.255G>C | p.Leu85Phe | 2.2 | 1.9 |
| c.200C>G | p.Pro67Arg | 2.2 | 1.0 |
| c.341T>C | p.Ile114Thr | 2.2 | 1.0 |
| c.335G>A | p.Cys112Tyr | 4.4 | - |
| c.319C>T | p.Leu107Phe | 4.4 | - |
| c.425G>C | p.Gly142Ala | 2.2 | 1.0 |
| c.418A>C | p.Asn140His | 2.2 | 1.0 |
| c.358-304C>G | pseudoexon inclusion | - | 1.9 |
| c.281G>T | p.Gly94Val | - | 1.9 |
| c.328G>T | p.Asp110Tyr | - | 1.9 |
| c.44T>G | p.Val15Gly | - | 1.9 |
| c.38G>C | p.Gly13Ala^a^ | - | 1.9 |
| c.339C>G | p.Ileu133Met | - | 1.9 |
| c.413C>T | p.Thr138Ile | - | 1.9 |
| c.281G>A | p.Gly94Asp | - | 1.9 |
| c.443G>A | p.Gly148Ala | - | 1.9 |
| c.376G>A | p.Asp126Asn | - | 1.9 |
| c.217G>A | p.Gly73Ser | 4.4 | - |
| c.450T>G | p.Ileu150Met | - | 1.9 |
| c.409A>T | p.Lys137Stop | - | 1.9 |
| c.199C>G | p.Pro67Ala | - | 1.9 |
| c.365A>G | p.Glu122Gly | 2.2 | - |
| c.124G>A | p.Gly42Ser | 2.2 | - |
| c.400_402del | p.Glu134del | 2.2 | - |
| c.399_401delGAA | p.Glu134del | 2.2 | - |
| c.301G>A | p.Glu101Lys | 2.2 | - |
| c.455T>C | p.Ile152Thr | - | 1.0 |
| c.115C>G | p.Leu39Val | - | 1.0 |
| c.400G>T | p.Glu134X | - | 1.0 |
| c.50G>C | p.Gly17Ala | - | 1.0 |
| c.223C>T | p.Pro75Ser | - | 1.0 |
| c.64G>C | p.Glu22Gln | - | 1.0 |
| c.443G>C | p.Gly148Ala | - | 1.0 |
| c.380T>C | p.Leu127Ser | - | 1.0 |
| c.355G>C | p.Val119Leu | - | 1.0 |
| c.346C>G | p.Arg116Gly | - | 1.0 |
| c.35A>G | p.Asp12Gly | - | 1.0 |
| c.443G>T | p.Gly148Val | - | 1.0 |
| c.464A>C | p.Ter155SerextTer6 | - | 1.0 |
| c.118A>G | p.Asn140Asp | - | 1.0 |
| c.20G>A | p.Cys7Tyr | - | 1.0 |
| c.88G>A | p.Val29Met | - | 1.0 |
| c.278A>T | p.Asp93Val | - | 1.0 |
| c.436G>A | p.Ala146Thr | - | 1.0 |
| c.397_399del | p.Glu133del | - | 1.0 |
| c.34G>T | p.Asp12Tyr | - | 1.0 |
| c.195T>A | p.Phe65Leu | - | 1.0 |
| c.106A>G | p.Ile36Val | - | 1.0 |
| c.449T>C | p.Ile150Thr | - | 1.0 |
| c.443G>A | p.Gly147Asp | - | 1.0 |
| c.241C>T | p.His81Tyr | - | 1.0 |
| c199C>G | p.Pro67Ala | 2.2 | - |
| c.137T>C | p.Phe46Ser | 2.2 | - |
| r.18_239del | p.Cys7_Arg80del | 2.2 | - |
| c.184G>C | p.Gly62Arg | 2.2 | - |
| c.217G>T | p.Gly73Cys | 2.2 | - |
| c.376G>C | p.Asp126His | - | 1.0 |
| c.443G>A | p.Gly148Asp | - | 1.0 |
| c.380dup | p.Leu127Phefs*7 | 2.2 | - |
| c.197A>G | p.Asn66Ser | 2.2 | - |
| c.13G>A | p.Ala5Thr | 2.2 | - |
| c.412A>G | p.Thr138Ala | - | 1.0 |
| c.112G>A | p.Gly38Arg | - | 1.0 |
| c.38G>A | p.Gly13Asp | - | 1.0 |
| c.19T>A | p.Cys7Ser | - | 1.0 |
| c.42_44delAGTinsGGG | p.Val15Gly^a^ | - | 1.0 |
| [a] multiple variants may be reported for the same patient  Tofersen cohort n=46; Historic cohort n=103 | | | |

# Table S3: Sociodemographic, and clinical characteristics of p.Asp91Ala variant ALS-SOD1 patients

|  | **Historical (n=8)** | **Tofersen (n=3)** |
| --- | --- | --- |
| **Demographics** | | |
| Female, n (%) | 4 (50%) | 3 (100%) |
| Age at symptom onset (Y), median (IQR) | 58 (12.3) | 54.6 (18.2) |
| Age at diagnosis, (Y),median (IQR) | 60.1 (13.1) | 59.6 (20.5) |
| **Disease characteristics at diagnosis** | | |
| Diagnostic delay (month), median (IQR) | 28.4 (24.4) | 62.8 (36.6) |
| ALS onset, n (%) |  |  |
| Lumbar | 7 (87.5) | 3 (100) |
| Respiratory | 1 (12.5) | 0 |
| ALSFRS-R score, median (IQR) | 37.5 (35.0 - 42.0) | 42 (37.0 - 43.0) |
| ALSFRS-R slope since onset, median (IQR) | 0.39 (0.34 - 0.56) | 0.18 (0.12 - 0.19) |
| ALSFRS-R progression n (%) |  |  |
| Slow | 1 (12.5) | 3 (100) |
| Intermediate | 6 (75.0) | 0 |
| Fast | 1 (12.5) | 0 |
| **Disease characteristics at baseline** | | |
| ALSFRS-R score, median (IQR) | 37.5 (34.2 - 42.0) | 32 (31.5 - 32.0) |
| ALSFRS-R slope since onset, median (IQR) | 0.37 (0.33 - 0.54) | 0.09 (0.08 - 0.14) |
| NFL levels p, median (IQR) | NA | 28.5 (26.7 - 31.6) |
| **Disease characteristics at T6** | | |
| ALSFRS-R score, median (IQR) | 40 (31.0 - 41.5) | 31 (31.0 - 31.5) |
| Missing | 1 | 0 |
| ALSFRS-R slope since BL, median (IQR) | 0.00 (-0.12 - 0.43) | 0.00 (0.00 - 0.08) |
| Missing | 1 | 0 |
| NFL levels p, median (IQR) | NA | 22.4 (17.5 - 24.3) |
| **Disease characteristics at T12** | | |
| ALSFRS-R score, median (IQR) | 38 (28.7 - 40.2) | 31 (31.0 - 31.0) |
| Missing | 0 | 1 |
| ALSFRS-R slope since BL, median (IQR) | 0.15 (-0.02 - 0.36) | 0.04 (0.02 -0.06) |
| Missing | 0 | 1 |
| NFL levels p, median (IQR) | NA | 19.9 (18.9 - 20.9) |
| Missing |  | 1 |

# Figure S1: Comparison of ALSFRS-R progression rate and score between the Tofersen and the Historical Cohort in the fast progressors population


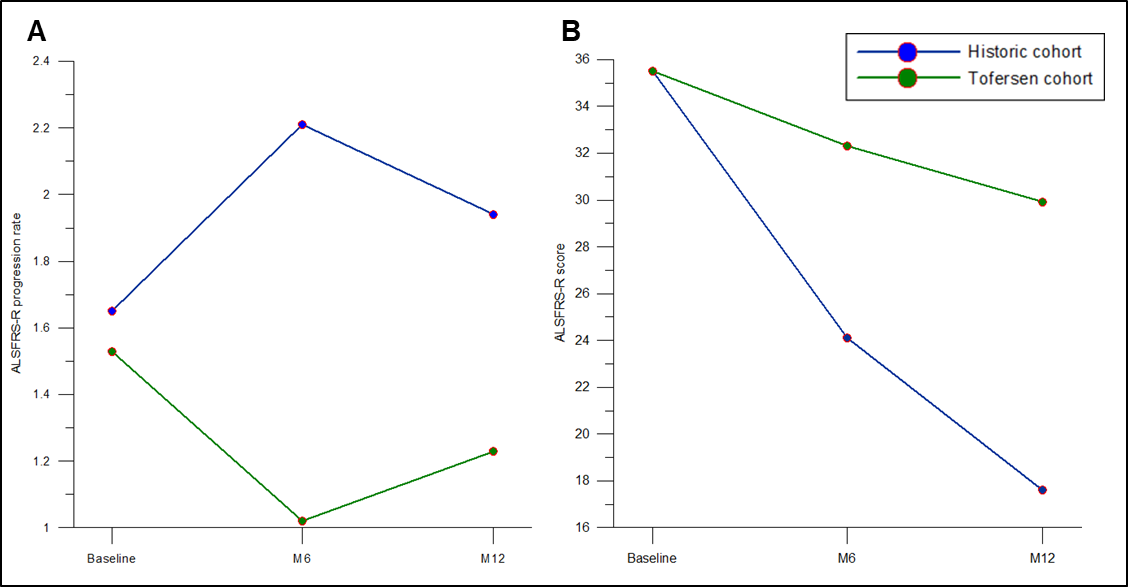


a. Estimated marginal means of the ALSFRS‑R progression at baseline, 6 and 12 months for the fast progressors in the Tofersen Cohort (N=14/12/10),and fast progressors in the Historical Cohort (N=37/21/13)

b. Estimated marginal means of the ALSFRS‑R score at baseline, 6 and 12 months for the fast progressors in the Tofersen Cohort (N=14/12/10), and fast progressors in the Historical Cohort (N=37/21/13).

ALS, amyotrophic lateral sclerosis; ALSFRS-R, ALS functional rating scale – revised; T6, 6 months post-treatment; T12, 12 months post-treatment

# Figure S2: Kaplan Meier analysis for the overall population


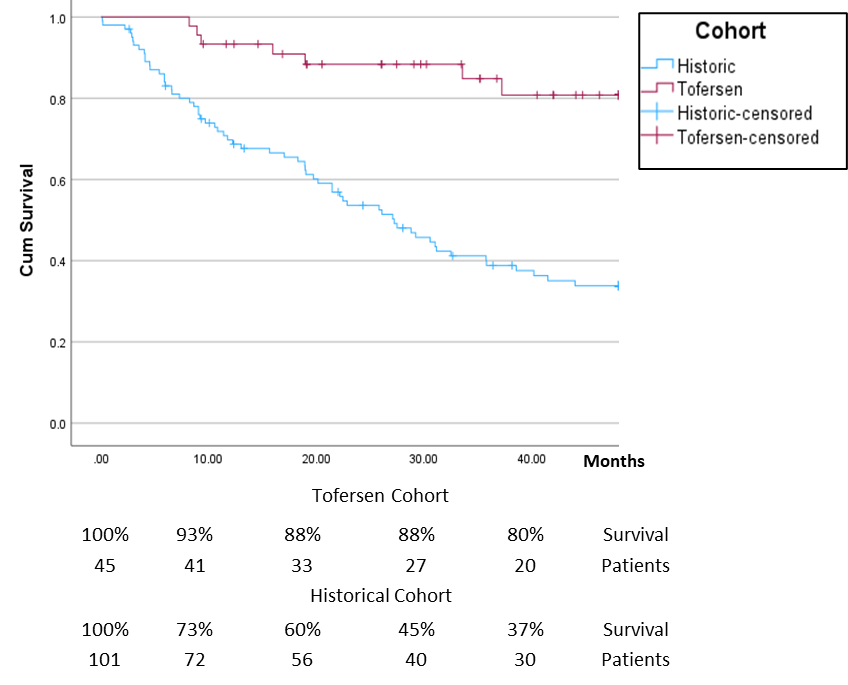


Kaplan–Meier survival curves after diagnosis, by cohort (Tofersen Cohort, n=45; Historical Cohort, n=101); Log‑rank test p<001; Holm-adjusted p=0.002.

# Figure S3: Kaplan Meier Analysis for the matched population


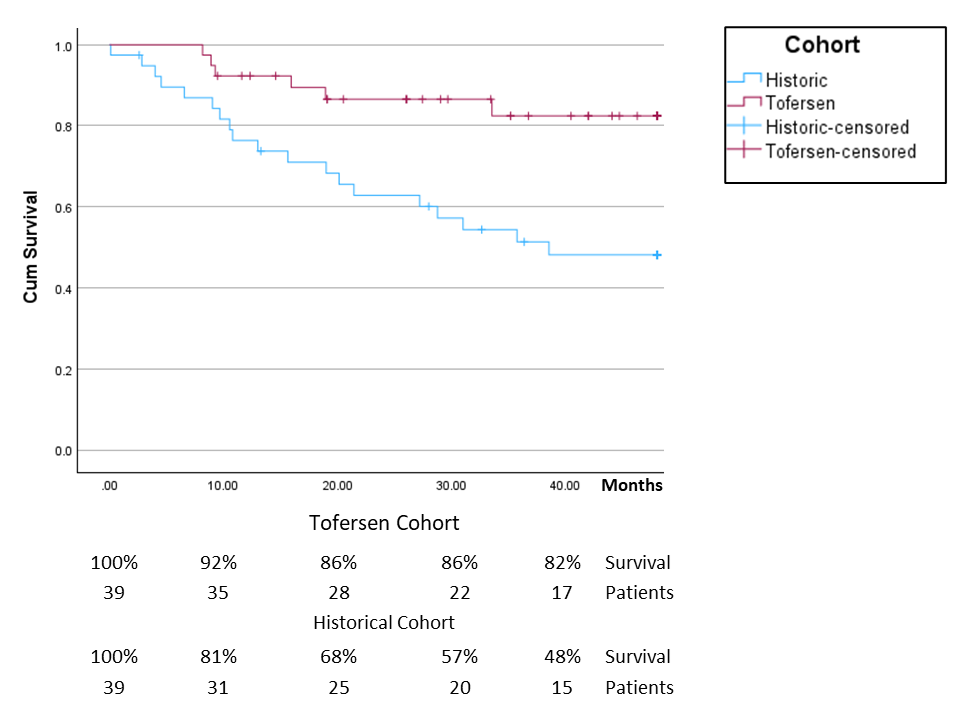


Matched population (Tofersen cohort n=39; Historical cohort n=39); Log‑rank test p=0.004; Holm-adjusted p=0.004.

# Table S4: Adjusted time-dependent Cox model.

|  | **Overall population** | | **Matched population** | |
| --- | --- | --- | --- | --- |
|  | **aHR (IC95%)** | **p value** | **aHR (IC95%)** | **p value** |
| Tofersen (time dependent variable) | 0.2 (0.09 – 0.5) | 0.001 | 0.34 (0.12 – 0.91) | 0.03 |
| Age at diagnosis | 1.0 (1.01 – 1.05) | 0.001 | 1.03 (1.00 – 1.07) | 0.02 |
| ALSFRS-R progression rate at diagnosis | 1.4 (1.05- 1.99) | 0.02 | 1.58 (1.01 – 2.45) | 0.04 |
| ALSFRS-R score at diagnosis | 0.9 (0.89- 0.98) | 0.007 | 0.9 (0.87-0.98) | 0.02 |
| Sex | 1.2 (0.7 – 2.1) | 0.42 |  |  |
| Diagnostic delay | 0.9 (0.9 – 1.0) | 0.10 |  |  |
| Site |  |  |  |  |
| Bulbar | 1 |  |  |  |
| Spinal | 0.4 (0.2 – 0.9) | 0.03 |  |  |
| aHR= adjusted hazard ratio |  |  |  |  |
